# Assessment of Saudi Females’ Knowledge Regarding Human Papillomavirus Infection, Screening, and Available Methods for Prevention: A Cross-Sectional Study in Qassim Region

**DOI:** 10.7759/cureus.33311

**Published:** 2023-01-03

**Authors:** Sohail A Alqarawi, Emad F Aljarbooa, Ahmed Y Almuqaytib, Ibrahim A Alomar, Mosaid H Altwaijri, Abdullah Y Aldakhil, Abdullah H Altowaijri

**Affiliations:** 1 Family and Community Medicine, Qassim Health Cluster, Buraydah, SAU; 2 General Surgery, Armed Forces Hospital Southern Region, Khamis Mushait, SAU; 3 College of Medicine, Qassim University, Buraydah, SAU; 4 College of Applied Sciences, Qassim University, Buraydah, SAU

**Keywords:** knowledge, screening, women, infection, human papillomavirus

## Abstract

Introduction

Human papillomaviruses (HPV) are known to be the main culprit of cervical cancer. It is the fourth most common cancer among women worldwide. In recent years, it has begun to spread more widely in Saudi Arabia. Saudi Arabia's Ministry of Health recently added HPV vaccination for women to the list of recommended vaccinations.

Aim

This study aimed to assess Saudi females' knowledge regarding HPV infection, screening, and the available tools for prevention in Qassim, Saudi Arabia.

Subject and methods

This is a descriptive cross-sectional study conducted among women living in the Qassim region of Saudi Arabia. A self-administered pre-structured questionnaire was distributed among the targeted women using an online platform. The questionnaire includes socio-demographic characteristics (i.e. age, education, occupation, etc.) knowledge questionnaire and a questionnaire about the attitude toward HPV protection. The minimum required sample size was 385. This means 385 or more measurements/surveys are needed to have a confidence level of 95% that the real value is within ±5% of the measured/surveyed value. All females aged 18 years and above who are living in the Qassim region of Saudi Arabia were included in our study. Males are excluded from this study. All data analyses were performed using Statistical Product and Service Solutions (SPSS) (IBM SPSS Statistics for Windows, Version 26.0, Armonk, NY).

Results

Of the 387 women involved, 52.2% were aged between 18 and 25 years old. The prevalence of women who have heard of HPV was 49.1%. Fifty-four percent were willing to accept HPV vaccination if offered. The overall mean knowledge score was 3.56 (SD 2.51) out of 11 points. Poor knowledge levels constituted most of the women (71.1%), 24.5% had moderate knowledge and only 4.4% were considered good. Increased awareness was more prevalent in younger women, more educated, those who underwent Pap smear, and those who were willing to accept HPV vaccination.

Conclusion

The awareness of women toward HPV infection was deficient. Younger women who had a better education and who are willing to receive HPV vaccination were more likely to demonstrate better awareness levels toward HPV infection as compared to the rest of the women. More research is needed to establish the level of awareness among women in our region.

## Introduction

Human papillomaviruses (HPV) are known to be the main culprit of cervical cancer, along with other diseases. They are mainly transmitted through sexual activity. HPVs are categorized into high-risk types and low-risk types. Low-risk genotypes (HPV 6 and 11) result in genital warts, a common benign condition of the external genitalia that leads to high morbidity. High-risk types 16 and 18 are responsible for 70% of cervical cancer and precancerous lesions.

Cervical cancer is ranked as the fourth most common cancer among women around the globe, with an estimated 604,000 new cases and 342,000 deaths in 2020 [1]. In Saudi Arabia, cervical cancer is the eighth most frequent cancer among women between the ages of 15 and 44, even though there are no national screening programs for the disease. It has become more prevalent in Saudi Arabia over the last few years. According to International Agency for Research on Cancer (IARC), about 358 new cases are reported annually of cervical cancer (estimates for 2021), with 179 deaths annually [2].

HPV vaccination was originally proposed for usage in the United States of America (USA) for female teenagers and youthful adults in 2006 and male teenagers and youthful adults in 2009 [3]. The presence of HPV vaccines that mainly protect against high-risk genotypes 16 and 18 was a huge step forward in reducing the burden of this disease [4]. According to the Centers for Disease Control and Prevention (CDC) statistics, since the beginning of HPV vaccines, they have been showing a huge effect in preventing HPV-related cancers and skin warts, reaching about 88% in young female teens and 81% among adult women [5].

The HPV vaccination has just been added to the official immunization schedule for women in the Kingdom of Saudi Arabia. However, the absence of national cervical cancer screening programs poses a significant health concern in the country, as early diagnosis of precancerous lesions by Pap screening is a major component in preventing cervical cancer [6]. Thereby, the importance of having adequate information about HPV is vital to prevent getting the infection that could lead to cervical cancer.

## Materials and methods

This was a descriptive cross-sectional study conducted among women living in the Qassim region of Saudi Arabia. A self-administered pre-structured questionnaire was distributed among the targeted women using an online platform. The questionnaire includes socio-demographic characteristics (i.e. age, education, occupation, etc.) knowledge questionnaire and a questionnaire about the attitude toward HPV protection. The minimum required sample size was 385. This means 385 or more measurements/surveys are needed to have a confidence level of 95% that the real value is within ±5% of the measured/surveyed value. All females aged 18 years and above who are living in the Qassim region of Saudi Arabia were included in our study, males are excluded from this study.

The knowledge of women regarding HPV has been assessed using an 11-item questionnaire with "yes" coded with 1 and “no/I don’t know” coded with 0 as the answer options. The total knowledge score has been calculated by adding all 11 items. A possible score range from 0 to 11 points had been generated. A higher score indicates higher knowledge levels of HPV. By using 50% and 75% cut-off for scores to determine the level of knowledge, participants were considered as having poor knowledge if the score was less than 50%, 50% to 75% were considered as moderate, and above 75% was considered a good knowledge level.

Descriptive statistics were used to describe the overall group of respondents including numbers and percentages (categorical variables), and mean and standard deviation (continuous variables). The differences in the scores of knowledge according to the socio-demographic and the attitude toward HPV protection had been conducted by using the Mann-Whitney Z-test. Statistical collinearity was measured using the Shapiro-Wilk test as well as the Kolmogorov-Smirnov test. The awareness score follows the non-normal distribution. Therefore, the non-parametric test was applied. Two-tailed analysis with p<0.05 was used as the cutoff for statistical significance. All data analyses were performed using Statistical Product and Service Solutions (SPSS) (IBM SPSS Statistics for Windows, Version 26.0, Armonk, NY).

## Results

This study involved 387 women. Table 1 presents the socio-demographic characteristics of the participants. A total of 52.2% were aged between 18 and 25 years old. Respondents with bachelor’s degree holders constitute 73.4%. Only 38.2% were employed and 53.5% were still single.

**Table 1:** Socio-demographic characteristics of women (n=387)

Study Data	N (%)
Age group	
18-25 years	202 (52.2%)
26-32 years	60 (15.5%)
33-39 years	31 (08.0%)
≥40 years	94 (24.3%)
Educational level	
High school or below	13 (03.4%)
Diploma	80 (20.7%)
Bachelor's degree	284 (73.4%)
Postgraduate	10 (02.6%)
Occupational status	
Employed	148 (38.2%)
Housewife/Unemployed	239 (61.8%)
Marital status	
Single	207 (53.5%)
Married	163 (42.1%)
Divorced or widowed	17 (04.4%)

Table 2 assessed women’s knowledge and attitudes about HPV. Based on the results, it was observed that the proportion of women who have heard of HPV was 49.1%. More than half (51.4%) were aware that HPV vaccination is not only for those who have the disease while 45.2% knew that a Pap smear can detect HPV. The proportion of women who disagreed that vaccination against HPV can replace the periodic examination was 42.9%. Only 34.6% of women believed that HPV can lead to cervical cancer and only 33.9% knew that HPV vaccination is safe. The prevalence of women who were aware of the availability of vaccination for HPV was 33.9%. Women showed a lack of awareness when asked if HPV can cause genital warts (21.4%) and only 17.3% disagreed that papillomavirus was intended for females only. In addition, only a few women (16%) believed that they should undergo regular screening every three years while only 10.3% knew that HPV infection cannot be detected in the blood. Based on the above statement, the overall mean knowledge score was 3.56 (SD 2.91) with 71.1% being considered as having a poor level of knowledge, 24.5% moderate, and only 4.4% considered as having good knowledge levels. Regarding the attitude toward HPV prevention, women who underwent Pap smears were only 18.6% and those who received HPV vaccination were only 2.1%. However, more than half (54%) were willing to accept HPV vaccination if given.

**Table 2:** Assessment of women’s knowledge and attitude about human papillomavirus (HPV) (n=387) HPV - human papillomavirus † indicates negative questions

Knowledge statement	Yes (%)
Heard of HPV	190 (49.1%)
Is vaccination against HPV given only to those who have had the disease? ^†^	199 (51.4%)
Can HPV be detected by a Pap smear?	175 (45.2%)
Does vaccination against HPV replace the periodic examination? ^†^	166 (42.9%)
Is HPV a cause of cervical cancer?	134 (34.6%)
Is the papillomavirus vaccination safe?	132 (34.1%)
Is there a vaccination for HPV?	131 (33.9%)
Is HPV one of the causes of genital warts?	83 (21.4%)
Is the papillomavirus vaccination for females only? ^†^	67 (17.3%)
Regular screening for the HPV is done every three years	62 (16.0%)
Can HPV infection be detected through blood? ^†^	40 (10.3%)
Total knowledge score (mean ± SD)	3.56 ± 2.91
Level of awareness	
Poor	275 (71.1%)
Moderate	95 (24.5%)
Good	17 (04.4%)
Attitude toward HPV prevention	
Have you ever had a pap smear?	72 (18.6%)
Did you get the HPV vaccination?	08 (02.1%)
If you were given the papillomavirus vaccination, would you accept it?	209 (54.0%)

When measuring the differences in the score of knowledge according to the socio-demographic characteristics and the attitude of women toward HPV protection (Table 3), it was found that a higher knowledge score was more associated with being younger in age (Z=3.198; p=0.001), more educated (Z=2.479; p=0.013), having undergone a Pap smear (Z=3.866; p<0.001), and willingness to received HPV vaccination (Z=7.341; p<0.001).

**Table 3:** Difference in the score of knowledge in relation to the socio-demographic characteristics and attitude of women toward HPV protection (n=387) § P-value has been calculated using Mann-Whitney Z-test ** Significant at p<0.05 level HPV - human papillomavirus

Factor	Knowledge Score (11) Mean ± SD	Z-test	P-value
Age group			
≤25 years	4.05 ± 3.09	3.198	0.001 **
>25 years	3.02 ± 2.59		
Educational level			
Diploma or below	2.86 ± 2.53	2.479	0.013 **
Bachelor or higher	3.79 ± 2.99		
Occupational status			
Employed	3.79 ± 2.80	1.387	0.166
Housewife/Unemployed	3.42 ± 2.97		
Marital status			
Never been married	3.78 ± 3.01	1.473	0.141
Been married	3.31 ± 2.78		
Ever had a pap smear			
Yes	4.78 ± 2.89	3.866	<0.001 **
No	3.29 ± 2.85		
Willingness to accept papillomavirus vaccination if given			
Yes	4.54 ± 2.79	7.341	<0.001 **
No/I don’t know	2.42 ± 2.62		

## Discussion

Our study evaluates the knowledge of women regarding HPV infection, screening, and prevention. The findings of this study showed that the overall knowledge of women about HPV infection and prevention was lacking. Approximately 71.1% were categorized as poor levels. A total of 24.5% were moderate and only 4.4% were categorized as having a good level of knowledge (mean score: 3.56; SD 2.91, out of 11 points). These findings are consistent with the literature wherein most women demonstrated a lack of understanding regarding the basic facts of HPV, infection, screening, and prevention [7-13]. In Aseer Region, Saudi Arabia, there has been a report that only a small proportion (17.4%) of Saudi females had good knowledge about cervical cancer [14].

The results of our study indicate that younger women (≤25 years) showed better knowledge levels than older ones (>25 years). This mirrored the study that was published in 2021, which reported that respondents who were younger (18 to 25 years) exhibited better knowledge of HPV [12]. Contradicting these reports, a study done in Madinah Region, Saudi Arabia, found that older women were more aware of HPV than younger ones. More investigations are required to determine the true effect of age in terms of awareness regarding HPV [10].

Increased awareness was more prevalent among educated women. This is consistent with the other two studies, both studies found education levels as one of the key factors for HPV awareness [15,16]. Furthermore, we also discovered that women who underwent Pap smear tests and those who were willing to accept HPV vaccination were seen to have better awareness levels compared to their counterparts.

The lack of awareness of our respondents stemmed from the specific questions related to the basic facts about HPV. For example, those who have heard of HPV were just almost half of our samples (49.1%), women who were aware that HPV can be detected through a Pap smear were also below half of our subjects (45.2%) and they had a poor understanding that HPV can lead to cervical cancer (34.6%) or can cause genital warts (21.4%) and relatively few women believed that HPV infection does not detect in blood samples (10.3%). In addition, only 16% were aware of the condition that HPV screening should be done regularly every three years. In a study done in Riyadh, Saudi Arabia, women showed a better awareness level that HPV was transmitted sexually (78.9%), or that it caused genital warts (81.7%) and cervical cancer (81.9%) [10]. However, contradicting these reports, stating that nearly half of the subjects (49%) disagreed that HPV is a common sexually transmitted infection and only 38% agreed that all cervical cancers are caused by HPV infection.

Moreover, we discovered that while more than half of our subjects (51.4%) disagreed that HPV vaccination is intended for those who had the disease, however, only 17.3% were against the opinion that the vaccination is for females only. Likewise, 42.9% viewed periodic examinations may have been better than HPV vaccination. Only one-third of the respondents believed the availability of HPV vaccines and the proportion of women who indicated its safety was suboptimal (33.9%).

Only 18.6% of our subjects had undergone Pap smear tests. We further observed that only 2.1% had ever gotten an HPV vaccination, although 54% showed a willingness to accept the HPV vaccine if offered. In Aseer Region, Saudi Arabia, a survey involving 1,116 showed a similar proportion of women had Pap smear test screening (27%) while in Makkah, Saudi Arabia a negative attitude was documented among married women, wherein the majority did not perform the Pap smear test regularly and most of them never had a PAP smear done adding that the lack of knowledge was the main reason for not having it [14,17].

The study's limitation was that it only obtained its information from the Qassim region. This gives us a more concentrated result as opposed to one that is more generalized. We recommend that in future research, data be collected from primary health centers, schools, and universities in a broader range of Saudi Arabia regions in order to ensure a more generalized result.

## Conclusions

The awareness of women toward HPV infection was deficient. Younger women who had a better education and who are willing to receive HPV vaccination were more likely to demonstrate better knowledge levels toward HPV infection as compared to the rest of the women. There is a need to improve awareness regarding HPV among women in our region. A more constructive program designed to bridge the gaps in knowledge is vital to achieving better understanding among the public. Awareness campaigns through social media, health educational campaigns, posters, pamphlets, and TV ads could provide a big boost to promote HPV infection, HPV vaccinations, and HPV screening programs. More research is needed probably at a national level with a bigger sample size to establish the level of awareness of women regarding HPV infection, screening, and prevention.

## Appendices

**Table 4:** Questionnaire

Do you agree to participate in the survey?		yes/no
Gender?		Male/Female
Age?		18-25 years/26-32 years/3-39 years/40 years and more
Educational level?		High school or under/Diploma/Bachelor degree/Postgraduate
Job status?		Employee/Housewife/currently not employee
Marital status?	Single/Married/Divorced/Widow	
Have you heard about the human papillomavirus?	Yes/No	
Is the human papillomavirus one of the causes of genital warts?	Yes/No/I do not know	
Is the human papillomavirus a cause of cervical cancer?	Yes/No/I do not know	
Can human papillomavirus infection be detected through blood?	Yes/No/I do not know	
Can human papillomavirus be detected by a Pap smear?	Yes/No/I do not know	
Have you ever had a pap smear?	Yes/No	
Regular screening for the human papillomavirus is done every three years :	Yes/No/I do not know	
Is there a vaccination for the human papillomavirus?	Yes/No/I do not know	
Did you get the human papillomavirus vaccination?	Yes/No	
If you were given the papillomavirus vaccination, would you accept it?	Yes/No/I do not know	
Does vaccination against the human papillomavirus replace the periodic examination?	Yes/No/I do not know	
Is the papillomavirus vaccination for females only?	Yes/No/I do not know	
Is vaccination against the human papillomavirus given only to those who have had the disease?	Yes/No/I do not know	
Is the papillomavirus vaccination safe?	Yes/No/I do not know	
